# Characterization of Six Complete Mitochondrial Genomes and ITS Sequences from *Armillaria mellea* (Vahl) P. Kumm.: A Phylogenetic Study and Comparative Analysis

**DOI:** 10.3390/ijms27083407

**Published:** 2026-04-10

**Authors:** Yuan Jiang, Yaping Li, Yuanfan Zhang, Jiadi Jin, Yisu Cao, Yanjun Wang, Zhirong Sun

**Affiliations:** School of Chinese Materia Medica, Beijing University of Chinese Medicine, Beijing 102488, China; 20220941431@bucm.edu.cn (Y.J.); 20240935178@bucm.edu.cn (Y.L.); 20210935141@bucm.edu.cn (Y.Z.); 20250935214@bucm.edu.cn (J.J.); 20250935215@bucm.edu.cn (Y.C.); 20220935153@bucm.edu.cn (Y.W.)

**Keywords:** mitochondrial genome, *Armillaria*, phylogenetic relationship, ITS, fungi

## Abstract

*Armillaria* species hold significant ecological and economic importance and they play a vital role in the growth of traditional Chinese medicine *Gastrodia elata* (*G. elata*). In this study, we assembled and compared the mitochondrial genomes (mitogenomes) of six *Armillaria mellea* (Vahl) P. Kumm. (*A. mellea*) strains isolated from the main *G. elata*-producing region of Hanzhong, China. The internal transcribed spacer (ITS) sequencing confirmed that all six strains form a monophyletic clade. Their mitogenomes (120,775 to 120,839 bp) exhibit a highly conserved architecture, each containing 16 protein-coding genes (PCGs), 23 open reading frames (ORFs), 27 tRNAs, and two rRNAs. Codon usage and amino acid frequency were strikingly similar among the six strains, with a strong AT bias. In contrast, comparisons with other *Armillaria* species revealed marked differences in gene order, repeat structures, and selection pressures. Phylogenetic analyses based on PCGs further resolved the close relationship among the six strains while highlighting distinct molecular variation across species. On the whole, these findings demonstrate that *A. mellea* strains co-evolving with *G. elata* maintain a highly uniform mitochondrial genome architecture, suggesting strong purifying selection or recent divergence within this symbiotic population. The pronounced differences from other *Armillaria* species at the levels of gene arrangement and selection pressure imply that mitochondrial gene rearrangement may have accompanied species diversification in the genus. By providing the first complete mitogenomes of *A. mellea* from a major *G. elata* cultivation area, this study not only expands the genomic resources for *Armillaria* but also establishes a foundation for understanding how mitochondrial variation might influence fungal growth, adaptation, and symbiotic efficiency with *G. elata*.

## 1. Introduction

*Armillaria*, (Basidiomycota, Physalacriaceae) [1] is widely distributed across various climates worldwide and impacts more than 500 hosts [2]. Eastern Asia represents the biogeographic region with the highest species richness [3]. *Armillaria* can provide ecological benefits and economic value [4], such as helping forest regeneration and promoting forest succession [5,6]. Moreover, it has rich nutrients and functional compounds, like enzymes and secondary metabolites, which have potential application value in industries such as food and pharmaceuticals [7,8,9]. *Armillaria* species can also form symbiotic associations with the orchid *Gastrodia elata* [10]. *Gastrodia elata* is a rare Chinese herbal medicine that has the effect of calming wind and relieving spasmosis, calming liver-yang, dispelling wind, and clearing collaterals [11]. *Gastrodia elata* is a rootless, leafless heterotrophic plant that requires the fungi of the *Armillaria* genus to provide nutrients, so *Armillaria* members, such as *A. mellea*, *A. gallica*, etc., play essential roles in the nutrient supply and growth modulation of *G. elata* [10,12]. Unfortunately, not all of the fungi of the *Armillaria* genus can exert a symbiotic effect. One of the important reasons is that fungi will gradually deteriorate as the number of generations increases, with their reproductive capacity declining [13]. In actual production, the fungi of the *Armillaria* genus are similar to other fungi [14]. Moreover, the compatibility between the fungi of the *Armillaria* genus in different regions is not consistent with that in the Qinba Mountain area; the research shows that there are 16 biological species (CBS) of *Armillaria* fungi in China. Mating experiments and verification using the Genome Consistency Phylogenetic Species Recognition Method (GCPSR) has shown that these species exhibit significant genetic differentiation and reproductive isolation in different geographical regions [15]. We collected six *Armillaria mellea* (Vahl) P. Kumm. strains from the Qinba Mountains, which show good symbiotic compatibility with *G. elata*. On the one hand, their biological characteristic differences from other regions’ fungi of *Armillaria* genus are still not fully understood. On the other hand, among the more than 40 species in the genus *Armillaria*, complete mitogenomes have been reported for only four species to date. In this study, four publicly available mitogenomes, namely *A. borealis* (MH407470; NC_042230), *A. gallica* (MH878687), *A. sinapina* (MH282847; NC_042229), and *A. solidipes* (MH660713; NC_042231) were included based on stringent selection criteria, in which only fully assembled and well-annotated complete mitogenomes were retained, while partial sequences, individual mitochondrial genes, and fragmented assemblies were excluded through manual curation. Consequently, sequencing and analyzing the mitogenomes of *Armillaria* species is of great significance. It not only provides essential data for phylogenetic and evolutionary studies of the genus but also offers genetic insights into the *Armillaria*–*Gastrodia elata* interaction, which is fundamental to sustainable *G. elata* production.

In biological identification, particularly in molecular systematics and DNA barcoding techniques, ITS (internal transcribed spacer) is the most frequently mentioned molecular marker. Essentially, they are specific DNA segments within the genome. Scientists use the sequencing of these segments to distinguish the identities of different species [16,17]. ITS, a non-coding region in the ribosomal RNA cluster, is valued for its high intraspecific conservation and high interspecific variability [18]. The ITS 1 and 2 with the 5.8S and the intergenic spacer 1 (IGS1) are common discrimination sequences in fungal molecular identification [19]. However, the mitogenomes of *Armillaria* have been little studied [20], limiting a complete understanding of the genetic characteristics and evolutionary histories of *Armillaria*. Mitochondria are semi-autonomous organelles that are primarily responsible for cellular respiration. Most eukaryotes have their mitogenomes, which are thought to have been derived from the ancestral member of the Alphaproteobacteria via endosymbiosis. As the “second genome” of eukaryotes, the mitogenomes have many characteristics different from the nuclear genome. In fungi, mitochondrial DNA (mtDNA) is circular or linear [21]. They show frequent DNA rearrangement events and high variability of gene order, although high synteny and conserved gene order are also present between closely related species [22].

In the present study, we aimed to characterize the mitogenomes of *A. mellea* and to investigate their evolutionary patterns within the genus. We hypothesized that, despite belonging to the same species, different strains of *A. mellea* may exhibit variation in mitogenome structure and evolutionary features. To test this, six strains were subjected to ITS sequencing to confirm their taxonomic identity, and phylogenetic analyses using Maximum Likelihood (ML) and Bayesian inference (BI) were performed to verify their placement within the genus. Their complete mitogenomes were further sequenced, assembled, and annotated. Comparative analyses were conducted on genome organization, repetitive elements, codon usage, and selection pressure to assess the conservation and divergence of these features. This study provides new insights into intraspecific variation and mitochondrial genome evolution in *A. mellea* and enriches genomic resources for the genus *Armillaria*.

## 2. Results

### 2.1. ITS Sequence Analysis

**Figure 1:**
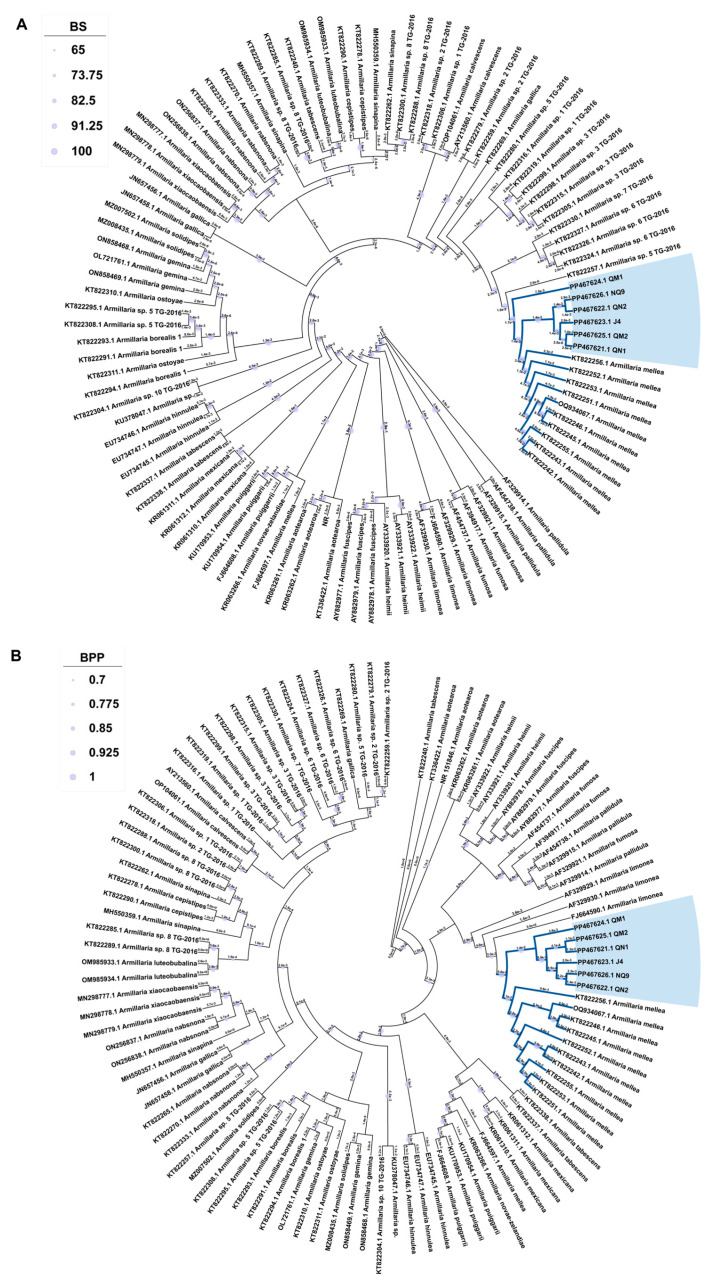
Molecular phylogeny of the ITS region in 102 *Armillaria* sequences based on ML (**A**) and BI (**B**) analyses. *Lentinus edodes* and *Ganoderma applanatum* were used as the outgroup. BS: Bootstrap, BPP: Bayesian posterior probability.

Based on the ITS sequences, the ML and BI phylogenetic trees were constructed to analyze the genetic relationship in the fungi of the *Armillaria* genus (Figure 1). All clades within the trees were supported (Bootstrap Value (BS) ≥ 65; Bayesian posterior probability (BPP) ≥ 0.7). The overall topology of the ML and BI trees was largely consistent with previously reported phylogenetic frameworks of *Armillaria* [23]. Major clades such as the *A. mellea*, *A. tabescens*, and *A. ostoyae*–*A. borealis* groups were recovered. The six strains formed a well-supported clade (BS = 100; BPP = 0.968) with short branch lengths (approximately 1.4 × 10^−3^ substitutions per site), indicating close genetic relationship among them. Then, they were clustered with the *A. mellea* strains downloaded from NCBI into the same branch, indicating that they were closely related. While the *A. mellea* clade is strongly supported, the lower thresholds for other internal nodes are displayed for topological context only and do not represent robust phylogenetic relationships.

### 2.2. Features and Protein-Coding Genes of the Armillaria Mitogenomes

**Figure 2:**
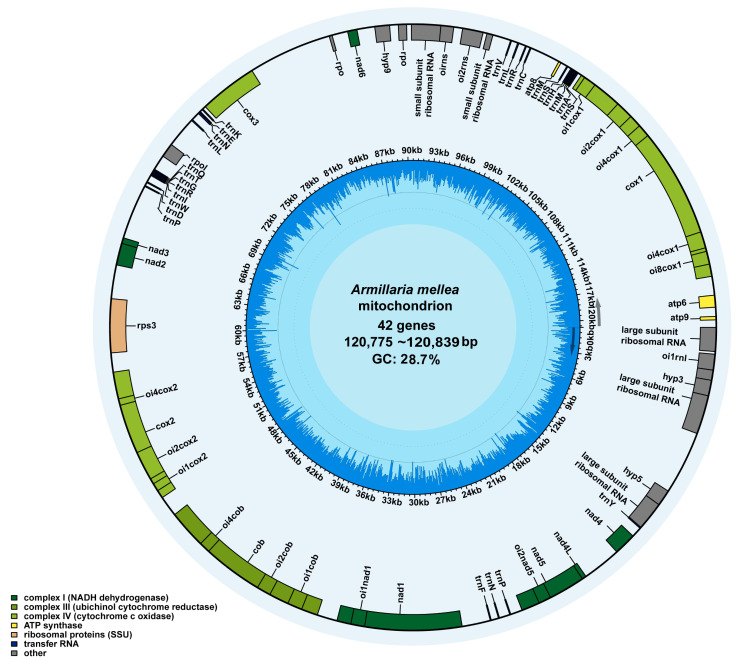
Mitogenome map of six strains of *Armillaria mellea*. Different colored blocks represent genes. Colored blocks within each ring indicate that the genes are on the direct strand.

The results of the reads mapping of the six mitogenomes showed uniform coverage and a non-zero value (Figure S1). The results indicated that the mitogenome assembly results of the six strains were correct and there was no heteroplasmy. All mitogenomes were reannotated using a consistent pipeline to ensure comparability, and homologous genes were identified based on sequence similarity. The size of the six mitogenomes of *A. mellea* was 120,779 bp (J4), 120,775 bp (NQ9), 120,839 bp (QM1), 120,785 bp (QM2), 120,789 bp (QN1), and 120,778 bp (QN2). The GC content of the six mitogenomes was 28.7%, and all PCGs, rRNAs, and tRNAs were located on the direct strand of the mitogenomes (Figure 2). These genomes were composed of 16 PCGs, 23 ORFs, 27 tRNA genes, and two rRNAs, for a total of 68 genes. In addition, the 23 ORFs are independent of the PCGs. Among them, the distribution of tRNA genes in the mitogenomes was clustered (Table 1). Mitochondrial genomes typically contain 15 core PCGs involved in oxidative phosphorylation. These include the mitochondrially encoded ATP synthase membrane subunits 6, 8, and 9 (*atp*6, *atp*8, *atp*9); cytochrome c oxidase subunits 1, 2, and 3 (*cox*1, *cox*2, *cox*3); cytochrome b (*cob*); NADH dehydrogenase subunits 1–6 (*nad*1, *nad*2, *nad*3, *nad*4, *nad*4 L, *nad*5, *nad*6); and a mitochondrial ribosomal protein S3 (*rps*3). Moreover, there were eight genes (*nad*5, *nad*1, *cob*, *cox*2, *cox*3, *cox*1, large subunit, and small subunit) that contained introns in the six mitogenomes. Among them, the types of introns were similar, and the *cox1* gene harbored the highest number of introns (Table S1). However, further analysis revealed that the other five strains were highly similar in all three dimensions (type, position, and encoded protein), whereas strain QM1 exhibited distinct intron-encoded proteins.

The six strains of *Armillaria mellea* share the same *rps3* gene length (3447 bp), which differs from those of four other known species: *A. borealis* (3438 bp), *A. solidipes* (2817 bp), *A. gallica* (3447 bp), and *A. sinapina* (3438 bp). *A. gallica* differs from the six strains in our study in terms of core PCG base composition and length. Specifically, it lacks 30 base pairs (bp) in the *atp*9 gene, while the *cob* gene in the six *A. mellea* strains is missing 252 bp. In the gene of *Armillaria solidipes* the highest number of bases are absent, specifically 621 bp.

### 2.3. Mitogenome Deletion Scale

**Figure 3:**
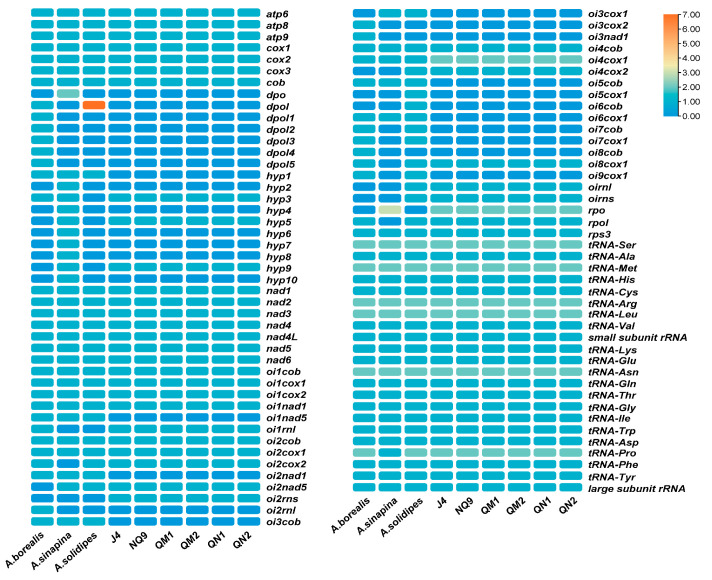
Gene deletion in the *Armillaria* genus. Each column represents a strain of *Armillaria*. Each row represents a gene. The grid color from red to blue represents copy numbers 7 to 0.

To explore the *Armillaria* species mitogenome deletion scale, we performed a heat map of gene deletion using *A. borealis* as a reference (Figure 3). The intronic ORFs deoxyribonucleic acid polymerase (*dpol*), *dpol*1, *dpol*2, *dpol*3, *dpol*4, *dpol*5, hypothetical proteins 1 (*hyp*1), open reading frame in intron 1 of NADH dehydrogenase subunit 5 gene (*oi1nad*5), *oi2nad*1, *oi2rn*l, *oi3cob*, *oi3cox*2, *oi3nad*1, *oi5cob*, *oi6cox*1, *oi7cob*, *oi7cox*1, *oi8cob* and *oi9cox*1 were lost in all *Armillaria* except *A. borealis*. Compared with the known complete mitogenomes of *A. borealis*, *A. sinapina*, and *A. solidipes*, six strains of *A. mellea* have a unique gene open reading frame in intron 2 of the small subunit ribosomal RNA gene (*oi2rns*), and two copies of the *oi4cox*1 gene, which are different from the known mitogenome of *Armillaria*. All species of *Armillaria* shown in Figure 2 had the 16 core PCGs, and the genes *oi1cob*, *oi1cox*1, *oi1cox*2, *oi1nad*1, *oi2cob*, *oi2cox*1, *oi4cob*, *oi4cox*1, and *rps*3, which is consistent with previous studies. Moreover, the number of tRNAs and rRNAs was the same for all species in Figure 2 except *A. sinapina* [24,25].

### 2.4. Codon Usage Analysis

**Figure 4:**
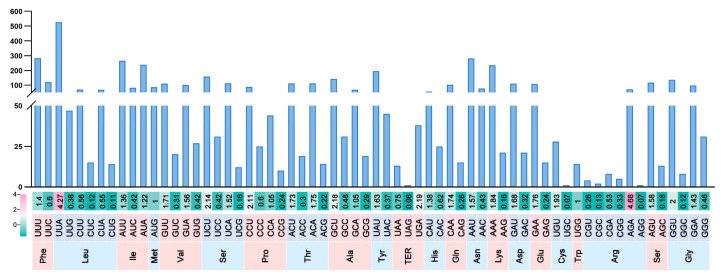
The RSCU values of all PCGs for six strains of *Armillaria mellea*. Color key: the red values indicate higher RSCU values and the green values indicate lower RSCU values.

Prior investigations have revealed that codon usage is directly related to the rate of translation and the energy required, and genes in mitochondria prefer to use specific codons to save time and energy for cell growth [25]. In this study, codon usage patterns of 16 PCGs from six strains of *A. mellea* mitogenomes were systematically analyzed. The Relative Synonymous Codon Usage (RSCU) results revealed a highly conserved codon usage pattern among the six strains, all showing a strong preference for AT-rich codons. A total of 22 amino acids (including stop codons) were identified. Specifically, codons ending with A or U were overwhelmingly favored, with UUA (526), UUU (283), and AAU (281) being the most frequently used, whereas GC-rich codons such as UGC (1), AGG (1), and UAG (1) were rarely utilized (Figure 4). This pattern indicates a pronounced AT bias in the mitogenomes of *A. mellea*. When comparing the codon usage patterns between *A. mellea* and the other four *Armillaria* species (*A. solidipes*, *A. sinapina*, *A. gallica*, and *A. borealis*), a high degree of conservation was observed. All species exhibited a pronounced bias toward AT-rich codons, with the most frequently used codons being nearly identical, including UUA (Leu), UUU (Phe), and AAU (Asn) in *A. mellea*, and UUA, UUU, AUA, AUU, and GGU in the other four species. This consistency suggests that the overall codon usage landscape is highly conserved within the genus *Armillaria*. Nevertheless, minor differences were noted. For instance, the codon CGC (Arg) was present but not preferred in *A. mellea*, whereas it was universally absent in the other four species [26]. Compared to the other species of Basidiomycota, the codon preference of five strains of *Armillaria*, except for QN1, was similar to that of *Ganoderma lucidum* and *Pleurotus*, which had high usage frequency in codon UUA [27,28]. Similar to other fungal studies, mitochondrial genes of six *A. mellea* strains had a high number of AT-rich codons, and similar codon frequencies are found in other fungal mitogenomes [27,28].

### 2.5. Repeat Analysis

**Figure 5:**
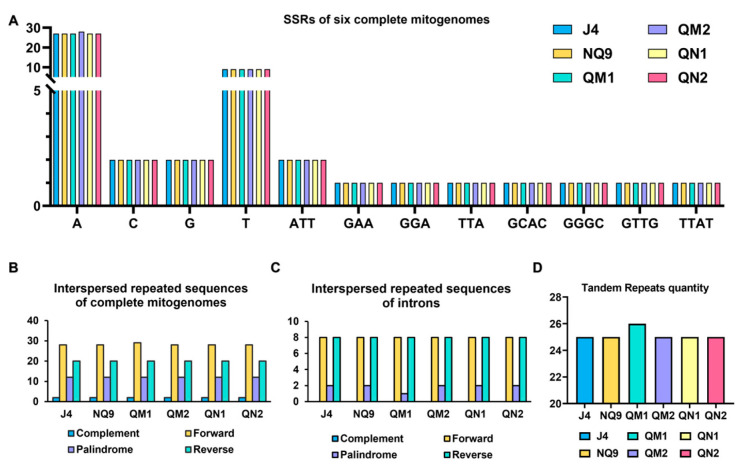
Comparison of repeats in the mitogenomes of six strains of *Armillaria mellea.* (**A**) SSRs for mitogenomes of six strains of *Armillaria mellea*. (**B**) Interspersed repeated sequences of complete mitogenomes. (**C**) Interspersed repeated sequences of introns. (**D**) Tandem repeats quantity.

Our analysis characterized the Simple Sequence Repeats (SSRs) in the mitogenomes of six *A. mellea* strains. The types of these SSRs were mononucleotide repeating units, trinucleotide repeating units, and tetranucleotide repeating units (Figure 5A). The numbers and types of SSRs in the six mitogenomes were similar, with 40–41 single-nucleotide repeats, five trinucleotide repeats, and four tetranucleotide repeats. The main type and number of mononucleotide repeats was A, and the trinucleotide repeats were ATT.

In addition, we also searched for tandem repeat loci using the Tandem Repeats Finder. A total of 26 tandem repeats were detected in the QM1 mitogenomes, and the other five mitogenomes had 25 repeats. The longest tandem repeat of QM1 was 64 bp, copied twice, and the longest sequence of the other five mitogenomes was 33 bp, also copied twice, with a resulting difference in repeat length (~62 bp) (Table S2). Among them, there was one tandem repeat from the large subunit rRNA exon and four tandem repeats from the large subunit rRNA introns. Moreover, the tandem repeats analysis of the GIY-YIG and LAGLIDADG sequences revealed that no tandem repeats exist in these sequences. Notably, QM1 exhibited a slightly larger mitogenome size (120,839 bp) compared to the other strains (120,775~120,789 bp), the length difference is approximately 60 bp and matches the observed difference in repeat length, suggesting that variation in tandem repeat length may contribute to genome size differences among strains.

Analysis of interspersed repeated sequences in the six strains’ mitogenomes revealed high similarity among them (Figure 5). Specifically, each mitogenome contained two complement repeats, 12 palindrome repeats, and 20 reverse repeats. Additionally, the QM1 mitogenome contained 28 forward repeats, while the other five strains’ mitogenomes contained 29 forward repeats. Among them, eight forward repeats and eight reverse repeats were found in six mitochondrial introns. Only QM1 contained one palindrome repeat, while the mitochondrial introns of the other five strains each contained two palindrome repeats. A statistical analysis was conducted on the interspersed repeated sequences in the GIY-YIG and LAGLIDADG sequences of the six strains’ mitogenomes. The results revealed that the GIY-YIG sequences from all six strains lacked interspersed repeated sequences. While the LAGLIDADG sequences of five strains’ mitogenomes contained one forward repeat in *cox*1, QM1 lacked it.

### 2.6. Comparative Mitogenomic Analysis

**Figure 6:**
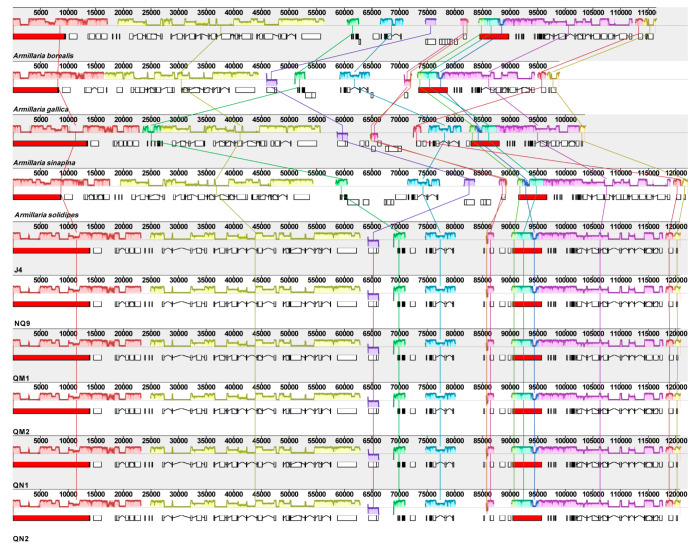
Collinearity of nine mitogenomes of *Armillaria*. The white rectangle represents CDS, the red rectangle represents rRNA, and introns are connected by line segments.

**Figure 7:**
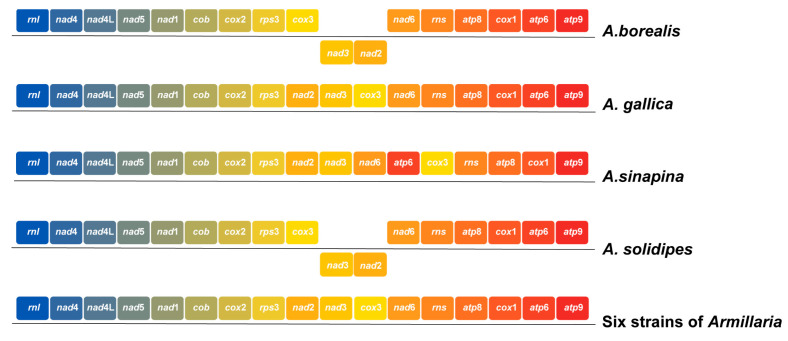
Core gene arrangement map. The same genes are indicated by squares of the same color. The squares located below the horizontal line correspond to the genes, which are situated on the reverse strand.

The collinear analysis showed that the six *A. mellea* mitogenome strains in this study share many homologous regions with other *Armillaria* (Figure 6). While some genes underwent genetic rearrangements and reversals, the position and size of genes changed significantly. The six *A. mellea* strains analyzed in this study showed complete collinearity without any gene rearrangement. Compared with the four species that have been publicly available in NCBI, *A. gallica* shows the highest degree of similarity in alignment with the six strains of *A. mellea*. Therefore, we discussed that within this species, the primary evolutionary consequence of these repeats is driving micro-scale length polymorphism (genome expansion) rather than large-scale structural recombination. Compared with the core PCGs of the other four *Armillaria* species, some genes showed rearrangements, such as *cox*3, *nad*2, *nad*3, *nad*6, *rns*, and *atp*6. Furthermore, the *nad*2 and *nad*3 genes occurred in reverse order in *A. borealis* and *A. solidipes* (Figure 7). Given that repetitive sequences frequently act as hotspots for homologous recombination in fungal mitogenomes, the accumulation and distribution of these repeats likely drive the large-scale structural rearrangements and evolutionary divergence observed among different *Armillaria* species.

### 2.7. Selective Pressure

**Figure 8:**
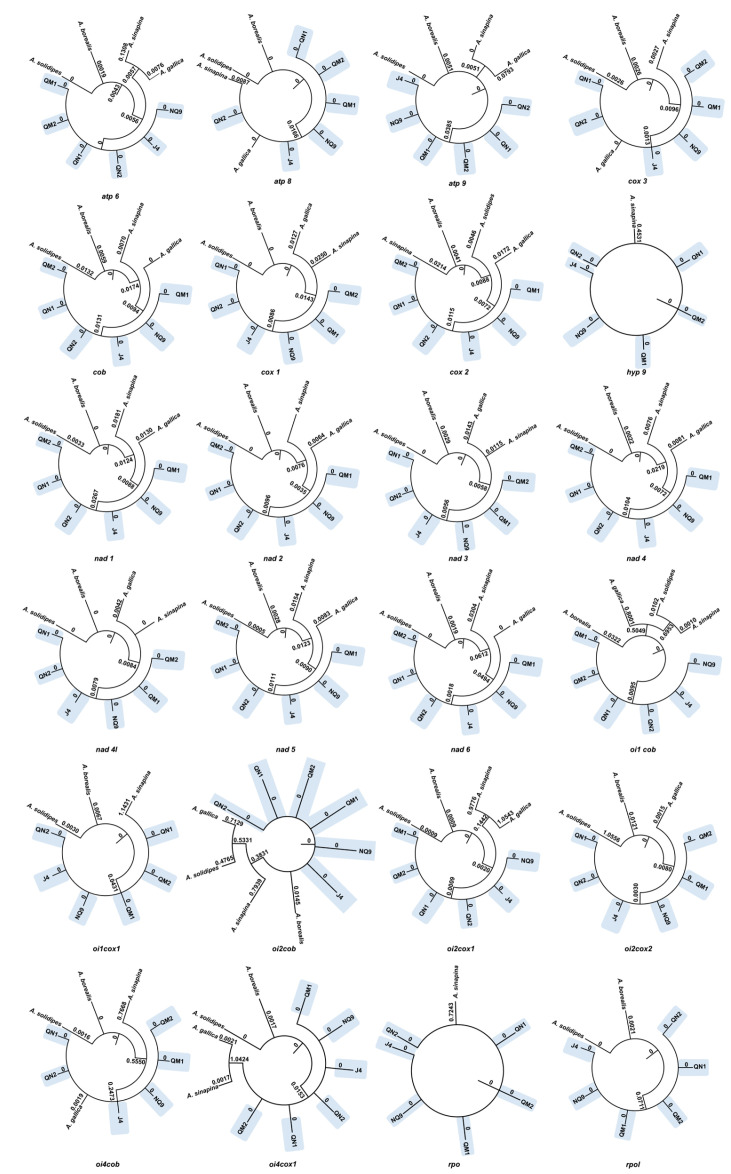
The dN/dS of 16 PCGs and ORFs (*oi4cox*1, *oi1cob*, *oi1cox*1, *oi2cox*1, *oi2cox*2, *oi4cob*, *oi2cob*, and *rpol*). The numbers on the branches indicate dN/dS (ω) value. The blue area represents the six strains of *A. mellea*.

We analyzed the selection pressure of 16 core genes. The ratio of non-synonymous to synonymous substitutions dN/dS (ω) values < 1 indicates that these genes were subject to purifying selection, while dN/dS (ω) values > 1 indicates that these genes were subject to positive selection. The result showed that the six strains of *A. mellea* had similarity in the *hyp9 gene* and *oi1cob*, *oi1cox1*, *oi2cox1*, *oi2cox2*, *oi4cob* ORFs; purifying selection occurred in all of them (Figure 8). In addition, positive selection occurred in some genes, for example *oi1cob* and *oi2cob* in *A. gallica* (MH878687), *A. sinapina* (MH282847; NC_042229), and *A. solidipes* (MH660713; NC_042231); *oi2cox1* and *oi4cox1* in *A. gallica* (MH878687) and *A. sinapina* (MH282847; NC_042229); *oi1cox1* and *oi4cob* in *A. sinapina* (MH282847; NC_042229); and *oi2cox2* in *A. solidipes* (MH660713; NC_042231).

### 2.8. Phylogenetic Analysis

**Figure 9:**
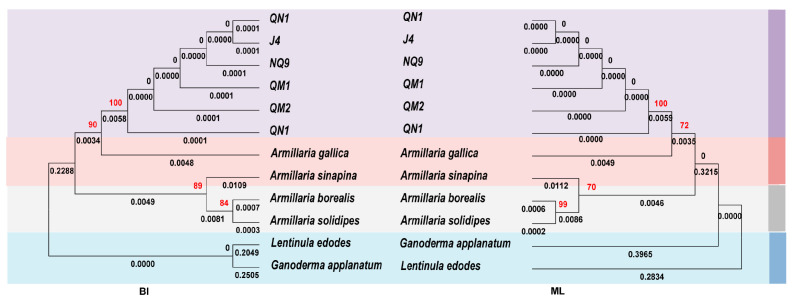
The ML (**right**) and BI (**left**) phylogenetic trees were constructed based on the 16 PCGs. *Lentinus edodes* and *Ganoderma applanatum* were used as the outgroup. The red data represents the support rate, while the black data represents the length of the evolutionary branch. The same color box indicates a closer genetic relationship.

The ML and BI phylogenetic trees were constructed based on the 16 PCGs to investigate the phylogeny of *Armillaria* (Figure 9). The mitochondrial phylogeny robustly supports the monophyly of the six strains (bootstrap = 90) relative to other taxa, supporting their monophyly. However, the relationships within this clade remain poorly resolved, with most internal nodes lacking statistical support (bootstrap = 0). Subsequently, these six strains of *A. mellea* clustered together with *A. gallica* (bootstrap support values = 100). Moreover, *A. sinapina*, *A. borealis* and *A. solidipes* clustered in a branch, and each branch contains higher supporting values (>70). This result indicated that compared to the published mitogenomes of the *Armillaria*, the genetic relationship of the six strains of *A. mellea* was close with *A. gallica*.

The ML and BI phylogenetic trees were constructed based on the 16 PCGs (Figure 9). All six *A. mellea* strains formed a well-supported monophyletic clade (bootstrap = 90/100) relative to the other *Armillaria* species and outgroup taxa. Within this clade, however, the branching order among the six strains was completely unresolved (bootstrap = 0 for all internal nodes), indicating that the phylogenetic relationships among these strains could not be reliably determined from the concatenated PCG data. The six *A. mellea* strains clustered together with *A. gallica* with high support (bootstrap = 100). Additionally, *A. sinapina*, *A. borealis*, and *A. solidipes* formed another well-supported clade (bootstrap > 70).

## 3. Discussion

In this study, we collected six *A. mellea* strains symbiotic with *G. elata* in the Qinba Mountains of Shaanxi Province. The ITSs of six *A. mellea* strains were sequenced, and ML and BI phylogenetic trees were constructed. The results showed that the local topological structure of the ML and BI trees were basically consistent with the previous study, such as *A. cepistipes* and *A. sinapina* being a sister group, as well as *A. ostoyae* and *A. borealis* [23]. Figure 1 showed that the six strains were close to other *A. mellea*. Therefore, this result confirmed that the six strains collected in this study were *Armillaria mellea*.

Mitogenomes provide high-resolution genetic data, which are instrumental in revealing intricate phylogenetic relationships and serve as an invaluable tool in the field of evolutionary biology. In this study, we first assembled and characterized six *A*. *mellea* mitogenomes, including their length, introns, gene arrangement, collinear analysis, and codon usage. Fungal mitogenomes are among the largest and most variable in length of eukaryotic mitogenomes. The size of complete fungal mitogenomes varies from the 12 kb mt genome of *Rozella allomycis* [29] to the 332 kb mt genome of *Golovinomyces cichoracearum* [30]. In the published complete *Armillaria* mitogenomes, their sizes are different (103, 563–122, 167 bp). *Armillaria solidipes* (MH660713; NC_042231) is similar in size to the mitogenomes in this study, about 120,000 bp [26]. Basidiomycota species generally contain 16 core PCGs, including 14 genes for energy metabolism (*atp*6, *atp*8, *atp*9, *cob*, *cox*l, *cox*2, *cox*3, *nad*l, *nad*2, *nad*3, *nad*4, *nad*4 L, *nad*5, and *nad*6) and one *rps*3 gene for transcriptional regulation [26]. In addition, 20–36 tRNA genes and two rRNA genes were also detected in basidiomycete mitogenomes [26]. The six mitogenomes in this study also contain the aforementioned 16 core PCGs, 27 tRNA genes, and two rRNA genes.

Introns are common in mitogenomes of fungi, and the number and type of introns vary greatly between different species. Moreover, they are considered one of the main factors contributing to variations in the size and organization of fungal mitogenomes [27,31]. Many mitogenomes contain introns in such genes as *cox1*, *cox2*, *cob*, *nad1*, *nad5*, and *rnl*. Table S1 shows that the six *A. mellea* strains in this study had the same total number of introns as *A. solidipes*. The *cox*1 gene was found to be the main gene of introns, representing 30% of all introns in the mitogenomes. Because the variation in introns in the *cox*1 gene could significantly affect the length and structure of the mitogenomes, the introns of *cox*1 were often selected as the object of intron research [32,33]. Most mitochondrial group I introns contain ORFs with GIY-YIG or LAGLIDADG homing endonuclease (HEGS) motif [31,34,35]. HEGs are one of the mobile genetic elements capable of inserting into specific genomic locations [36]. Kolesnikova et al. [26] found that *A. sinapina* contains 12 LAGLIDADG and seven GIY-YIG, *A. borealis* contains 15 and nine, *A. solidipes* contains 17 and eight, and *A. gallica* contains 13 and four. In this study, the *cox*1 of the five strains of *Armillaria* fungi revealed one LAGLIDADG. This striking reduction in intron number suggests a relatively simplified mitochondrial genome structure in *A. mellea* compared to other *Armillaria* species. Given that LAGLIDADG and GIY-YIG elements are typically associated with mobile introns, their variation is often linked to intron gain and loss events during evolution. Therefore, the low intron content observed here may reflect either intron loss or limited intron invasion, potentially contributing to the reduced genetic variation and weak phylogenetic resolution observed among the strains. Moreover, intron dynamics are known to play an important role in shaping fungal mitochondrial genome evolution. The reduced number of mobile introns in *A. mellea* may indicate a more conserved mitochondrial architecture, which could partly explain the limited sequence divergence detected in the phylogenetic analysis. Further comparative studies across *Armillaria* species will be necessary to clarify the evolutionary mechanisms underlying intron diversity and their impact on mitochondrial genome evolution.

Purifying selection typically acts to conserve existing phenotypes, whereas positive selection, also known as Darwinian selection [37], drives the emergence of novel adaptive traits, thereby enhancing a population’s fitness in its environment. In this study, several genes involved in oxidative phosphorylation within the mitogenomes of six strains of *A. mellea* were found to be under purifying selection. However, positive selection occurs in the *cox* and *cob* genes of *A. gallica*, *A. solidipes*, and *A. sinapina*, which may suggest adaptive modifications in oxidative phosphorylation. In high-altitude ecosystems, fungal species face severe environmental constraints such as hypoxia and low temperatures. Recent studies have demonstrated that positive selection and adaptive modifications often occur in key mitochondrial genes (e.g., *cox* and *cob*) of these fungi, ensuring efficient oxidative phosphorylation (OXPHOS) and robust ATP production under low oxygen availability [38]. Notably, these mitogenomic adaptations significantly impact their interactions with plant hosts. The modified OXPHOS machinery not only provides the massive energy required for successful host colonization and invasion but also modulates the fungal redox homeostasis, enhancing the fungus’s ability to scavenge or withstand the reactive oxygen species (ROS) bursts triggered by the plant immune system [39]. These changes may enhance ATP production efficiency and oxygen utilization under environmental constraints such as low oxygen availability at high altitudes. Given the obligate symbiotic relationship between *Gastrodia elata* and *Armillaria*, such mitochondrial adaptations in the fungus likely contribute indirectly to the ecological fitness of the host plant. Furthermore, the optimization of respiratory enzyme performance within a moderate temperature range (20–25 °C) may explain the observed growth preference of the symbiotic system.

The lack of phylogenetic resolution among the six *A. mellea* strains (bootstrap = 0) suggests that the mitochondrial PCG sequences do not contain sufficient phylogenetic signal to resolve their branching order. This could be due to a recent divergence event, low substitution rates in mitochondrial genes among these strains, or limited sequence variation within the analyzed genomic regions. Further analyses using higher-resolution markers (e.g., single-nucleotide polymorphisms from whole mitogenomes or nuclear markers) would be needed to clarify the relationships among these strains.

## 4. Materials and Methods

### 4.1. Sample Collection and Material Cultivation

**Figure 10:**
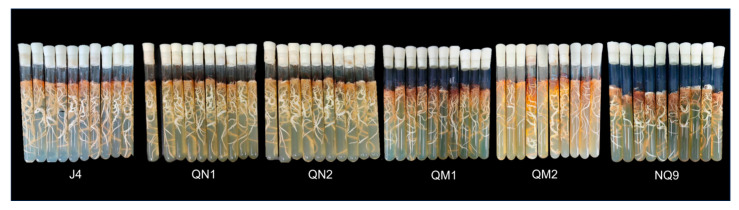
Six strains of *Armillaria mellea*.

Six strains of *A. mellea* were collected from Ningqiang County (Qinba Mountains), Hanzhong City, Shaanxi Province, China, in October 2022 (Figure 10). The *A. mellea* specimens and the wood to which they were attached were placed in an ice box and brought to the laboratory for preservation at 4 °C. Collect the black strands that are attached to the surface of the wood, sterilize the surface of the strands with ethanol (70%) for 30 s, and rinse three times in sterile water to avoid unnecessary contaminants from the surface. Inside the ultra-clean bench, use a scalpel to incise the black epidermis to expose the enclosed white hyphae. Finally, inoculate the uncontaminated white hyphae into test tubes filled with PDA cylindrical semi-solid culture mediums, with an inoculation length of 0.5 cm per tube. Place the inoculated test tubes in an environment at 24 °C and conduct dark cultivation for 20 days.

### 4.2. DNA Extraction and Sequencing

The DNA of six strains of *A. mellea* was extracted using the first generation with the Ezup Column Fungi Genomic DNA Purification Kit (Sangon Biotech, Shanghai, China). Then, internal transcribed spacer (ITS) regions of ribosomal RNA genes were amplified with the primers ITS1 (TCCGTAGGTGAACCTGCGG) and ITS4 (TCCTCCGCTTATTGATATGC [18]. Additionally, 1% agarose gel and 1 × TAE electrophoresis buffer solution were prepared. Electrophoresis (150 V, 100 mA) was performed for 20 min. The gel imager was for observation. The molecular weight shown by the bands was consistent with the expectation (900 bp), and the bands were clear, bright, and free of impurities. Then, PCR amplicons were subjected to bidirectional Sanger sequencing on an ABI 3730 XL instrument (Applied Biosystems, Thermo Fisher Scientific, Delaware, DE, USA) [40].

Mitogenomes sequencing libraries were constructed from the extracted genomic DNA using NEBNext Ultra II DNA Library Prep Kits (NEB, Beijing, China), following the manufacturer’s instructions. Whole genomic sequencing was performed on an Illumina HiSeq 2500 Platform and 250 bp paired-end reads were generated (6 Gb raw data, San Diego, CA, USA).

### 4.3. Quality Check, Assembly and Annotation of the Mitogenomes of Armillaria

The pair-end reads were trimmed for adapter and low-quality reads (Phred score < 30) using the NGS QC Toolkit v.2.3.3 software [41], resulting in the clean data being used for subsequent analysis. The six mitogenomes were assembled using NOVOPlasty V.3.8.3 (*A. gallica* MH878687 as seed sequence) [42]. The Bowtie2: V2.3.2 and Samtools v1.0 [43,44,45,46] were used to map the reads to the assembled genome and evaluate the effectiveness of the assembly results. Gaps between contigs were filled using MITObim V.1.9 [47]. Conducted coverage checks on six strains to illustrate the completeness of genome assembly and the uniformity of sequencing depth were then annotated by GeSeq version 2.03 [48] (https://chlorobox.mpimp-golm.mpg.de/geseq.html (accessed on 15 June 2025)). Annotation of mtDNA was performed using Geneious V.9.1.4 (https://www.geneious.com (accessed on 16 June 2025)), with the NCBI Reference Sequence: NC042230. To ensure comparability, all mitogenomes were re-annotated using a consistent pipeline, and homologous genes were identified based on sequence similarity and conserved annotation features.

### 4.4. Characterization and Comparative Analysis of Mitogenomes

The primary characteristics of the mitogenomes of six strains of *A. mellea* were analyzed. The total length of mitogenomes, number of genes, and base content were calculated by Geneious V.9.1.4 (https://www.geneious.com (accessed on 16 June 2025)); the length and number of introns were also counted by GeSeq [48]. Whole mitogenome alignments to clarify collinearity in the mitogenomes of six strains of *A. mellea* were performed using Mauve version v1.0 [49].

### 4.5. Repeat Analysis

Misa.pl version 1.0 was used to screen the simple sequence repeats (SSRs) [50]. Tandem repeats within the mitogenomes were also clarified using the Tandem Repeats Finder [51]. The interspersed repeated sequences of the mitogenome and introns were clarified using Reputer in (https://bibiserv.cebitec.uni-bielefeld.de/reputer (accessed on 20 June 2025)) [52].

### 4.6. Codon Usage

As in the research methods used in the previous study by the reference research group [53], the PCGs were extracted with Phylosuite V.1.2.2 [54]. RSCU and codon usage values were analyzed with CodonW V.1.4.2. The RSCU values were shown in a heatmap by Tbtools V.2.360 [55].

### 4.7. Selective Pressure in Armillaria Mitogenome Genes

The PCGs and ORFs were extracted with Phylosuite V.1.2.2 [54] and aligned with MAFFT v7 [56]. The above selection pressure of *Armillaria* was analyzed using an ML tree from the adaptive branch-site random effects likelihood (aBSREL) model in HyPhy. Nodes with *p* < 0.05 were considered to indicate that different selective pressures influence the sequence evolution of the mitogenome gene.

Codon-based alignments of PCGs were generated using MAFFT. Selection pressures were estimated using the aBSREL model in HyPhy v2.5 [57], which uses AICc to infer branch-specific ω rate categories. Likelihood ratio tests compared full (ω ≥ 0) vs. null (ω ≤ 1) models, with *p*-values corrected by Holm–Bonferroni. Branches with corrected *p* < 0.05 were considered to exhibit episodic positive selection (sites with ω > 1). All branches were tested.

### 4.8. Phylogenetic Analysis

The 102 published *Armillaria* rDNA-ITS sequences were downloaded from NCBI. Then, we aligned all rDNA-ITS using MAFFT v7 [56]. Constructed Maximum Likelihood (ML) and Bayesian inference (BI) phylogenetic trees were created using PhyloSuite 1.2.2 [54] with default parameter settings, and we visualized the trees using MEGA version X [57].

Mitogenome phylogenetic analysis was performed based on 12 complete mitogenomes, including the six assembled sequences in our study, and four mitogenomes downloaded from the NCBI database: *A*. *borealis* (MH407470, NC_042230), *A. gallica* (MH878687), *A. sinapina* (MH282847, NC_042229), *A. solidipes* (MH660713, NC_042231), *Lentinula edodes* (MF774813) and *Ganoderma applanatum* (KR109212) as outgroup. A total of 16 shared PCGs were extracted by PhyloSuite V.1.2.2 [54] and aligned using MAFFT v7 [56]. Subsequently, the alignment was conducted based on BI in MrBayes using the GTR  +  I  +  G evolution model [58]. The parameter was set to run for five million generations and sampled every 1000 generations, with all other settings left at their defaults, and the first 25% of each run was discarded as burn-in. The alignment was evaluated using bootstrap analysis on 1000 generations in a ML by RAxML [59], with parameters: raxmlHPC-PTHREADS-SSE3-fa-N1000-m GTRGAMMA-x551,314,260-p551,314,260-o Lentinula_edodes_MF774813, Ganoderma_applanatum_KR109212-T 20.

Finally, HyPhy v2.5 [60,61] was used for visualization. The screening criterion for the results was significance, with *p* < 0.05. HyPhy is an open-source software capable of analyzing selection pressure, providing various calculation models such as aBSREL, GARD, SLAC, FEL, FUBAR, and FADE. In this paper, the aBSREL [62] model was adopted. Through the branch-site model and likelihood ratio test, it was determined whether certain branches had undergone positive selection.

## 5. Conclusions

In this study, we constructed phylogenetic trees using *ITS* sequences to determine the phylogenetic position of six strains of *A. mellea* within the genus *Armillaria*. Subsequently, we assembled and characterized the mitogenomes of six strains of *A. mellea*. Compared with the four published mitogenomes, the gene types of the six mitogenomes were consistent. However, the PCGs of these mitogenomes exhibit rearrangements, and the number of repetitive sequences and the results of selective pressure analysis were different. The codon usage and amino acid frequency among the six strains of *A. mellea* showed a high similarity; both contain high AT content. The phylogenetic trees constructed by PCGs in the mitogenomes showed that these six mitogenomes form a monophyletic branch. Our study supplemented the biological information of *Armillaria mellea* mitogenomes and enhanced the genomic resources available for *Armillaria*.

## Figures and Tables

**Table 1:** Genes in the mitogenomes of six strains of *Armillaria mellea*.

Group of Genes	Gene Names	Amount
Protein-coding genes (PCGs)	*nad1*, *nad2*, *nad3*, *nad4*, *nad4 L*, *nad5*, *nad6*, *cob*, *cox1*, *cox2*, *cox3*, *atp6*, *atp8*, *atp9*, *rps3*, *rpo*	16
Intronic ORFs	*GIY-YIG endonuclease and intron ORFs (oi1cox1*, *oi2cox1*, etc.)	20
Intergenic ORFs (hypothetical proteins)	*hyp3*, *hyp5*, *hyp9*	3
rRNA	*rnl* (large subunit rRNA), *rns* (small subunit rRNA)	2
tRNA	Tyrosine, Proline (×2), Asparagine (×2), Phenylalanine, Aspartic acid, Tryptophan, Isoleucine, Arginine (×2), Glycine, Threonine, Glutamine, Leucine (×2), Glutamic acid, Lysine, Valine, Cysteine, Methionine (×3), Serine, Histidine, Alanine, Serine	27

## Data Availability

All sequences used in this study were submitted to NCBI with the accession numbers PP467623 (J4), PP467626 (NQ9), PP467624 (QM1), PP467625 (QM2), PP467621 (QN1), and PP467622 (QN2). The dataset generated and or analyzed during the current study is deposited in Genbank with the accession numbers OR885309 (J4), OR885310 (NQ9), OR885311 (QM1), OR885312 (QM2), OR783479 (QN1), and OR885313 (QN2).

## Supplementary Materials

The following supporting information can be downloaded at: https://www.mdpi.com/article/10.3390/ijms27083407/s1.

## Abbreviations

The following abbreviations are used in this manuscript:

*G. elata*	*Gastrodia elata*
ITS	Internal Transcribed Spacer
*A. mellea*	*Armillaria mellea*
PCGs	Protein-Coding Genes
ORFs	Open Reading Frames
*A. gallica*	*Armillaria gallica*
GCPSR	Genome Consistency Phylogenetic Species Recognition Method
*A. borealis*	*Armillaria borealis*
*A. sinapina*	*Armillaria sinapina*
*A. solidipes*	*Armillaria solidipes*
IGS1	Intergenic Spacer 1
mitogenomes	Mitochondrial Genomes
ML	Maximum Likelihood
BI	Bayesian Inference
BS	Bootstrap Values
BPP	Bayesian Posterior Probability
*A. tabescens*	*Armillaria tabescens*
*A. ostoyae*	*Armillaria ostoyae*
*Tef1-**α*	*Translation elongation factor 1-alpha*
*atp*6, *atp*8, *atp*9	*ATP synthase subunit* 6, 8, 9
*cox*1, *cox*2, *cox*3	*cytochrome c oxidase subunit* 1, 2, 3
*cob*	*cytochrome b*
*nad*1, *nad*2, *nad*3, *nad*4, *nad*4 L, *nad*5, *nad*6	*NADH dehydrogenase subunit* 1–6
*rps*3	*ribosomal protein S*3
*hyp*3, *hyp*5, *hyp*9	*hypothetical protein* 3, 5, 9
*dpol*, *dpol*1, *dpol*2, *dpol*3, *dpol*4, *dpol*5	deoxyribonucleic acid polymerase 1, 2, 3, 4, 5
*oilrnl*/*oirns*	*open reading frame in intron of the mitochondrial large/small ribosomal RNA*
RSCU	Relative Synonymous Codon Usage
SSRs	Simple Sequence Repeats
dN/dS	Ratio of Nonsynonymous Substitutions to Synonymous Substitutions
*A. cepistipes*	*Armillaria cepistipes*
HEGs	Homing Endonuclease
OXPHOS	Oxidative Phosphorylation
ROS	Reactive Oxygen Species
aBSREL	Adaptive Branch-Site Random Effects Likelihood
