# Supplementary figures and images for: Characterization of Six Complete Mitochondrial Genomes and ITS Sequences from Armillaria mellea (Vahl) P. Kumm.: A Phylogenetic Study and Comparative Analysis
Source: Int J Mol Sci. 2026 Apr 10;27(8):3407. doi: 10.3390/ijms27083407 (PMC13116349; doi:10.3390/ijms27083407)

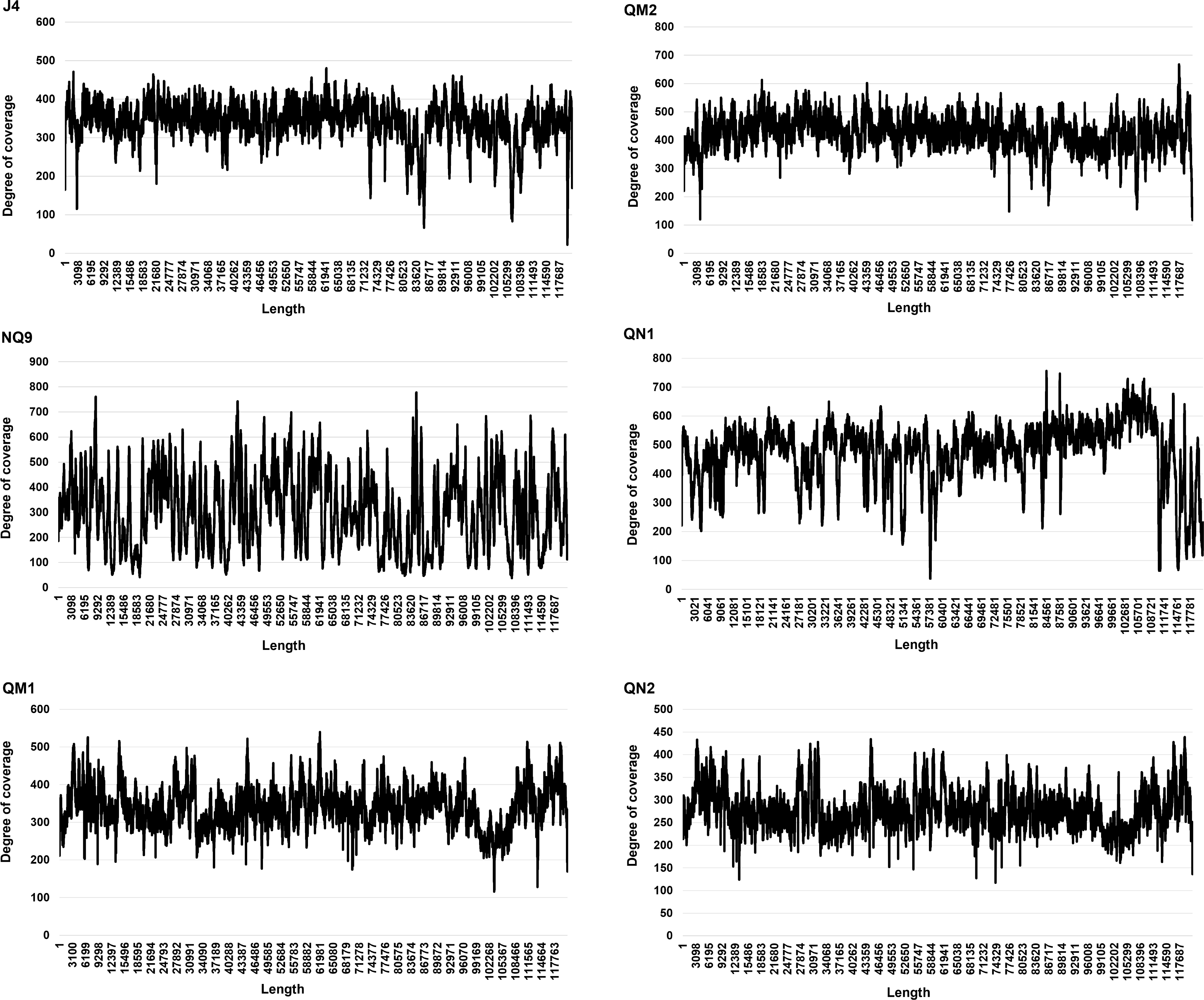

Supplement: Supplementary file 1 [file ijms-27-03407-s001.zip › Figure S1.tif]
